# All Roads Lead to Rome: Enhancing the Probability of Target Attainment with Different Pharmacokinetic/Pharmacodynamic Modelling Approaches

**DOI:** 10.3390/antibiotics12040690

**Published:** 2023-04-01

**Authors:** Kashaf Khalid, Katharina Rox

**Affiliations:** 1 Department of Chemical Biology, Helmholtz Centre for Infection Research (HZI), Inhoffenstraße 7, 38124 Braunschweig, Germany; kashaf.khalid@helmholtz-hzi.de; 2 German Center for Infection Research (DZIF), Partner Site Hannover-Braunschweig, 38124 Braunschweig, Germany

**Keywords:** target attainment, PK/PD, modelling, cefiderocol, ceftazidime, avibactam, gepotidacin, zoliflodacin, omadacycline

## Abstract

In light of rising antimicrobial resistance and a decreasing number of antibiotics with novel modes of action, it is of utmost importance to accelerate development of novel treatment options. One aspect of acceleration is to understand pharmacokinetics (PK) and pharmacodynamics (PD) of drugs and to assess the probability of target attainment (PTA). Several in vitro and in vivo methods are deployed to determine these parameters, such as time-kill-curves, hollow-fiber infection models or animal models. However, to date the use of in silico methods to predict PK/PD and PTA is increasing. Since there is not just one way to perform the in silico analysis, we embarked on reviewing for which indications and how PK and PK/PD models as well as PTA analysis has been used to contribute to the understanding of the PK and PD of a drug. Therefore, we examined four recent examples in more detail, namely ceftazidime-avibactam, omadacycline, gepotidacin and zoliflodacin as well as cefiderocol. Whereas the first two compound classes mainly relied on the ‘classical’ development path and PK/PD was only deployed after approval, cefiderocol highly profited from in silico techniques that led to its approval. Finally, this review shall highlight current developments and possibilities to accelerate drug development, especially for anti-infectives.

## 1. Introduction

The COVID-19 pandemic, which is currently moving towards the endemic phase in some parts of the world, has received great attention since 2020. However, out of the spotlight of COVID-19, the ‘silent pandemic’ of antimicrobial resistance (AMR) is still on-going and growing [1]. Back in 2014, the World Health Organization (WHO) already tried to put the spotlight on potential pathogens and defined a pathogen priority list [2]. Among the pathogens listed were the so-called *ESKAPE* pathogens [3], the *Enterococcus faecium*, *Staphylococcus aureus*, *Klebsiella pneumoniae*, *Acinetobacter baumannii, Pseudomonas aeruginosa*, and *Enterobacter* species [4]. 

The research landscape has remained relatively unchanged despite the priority list being in place for nearly a decade. The reason for this is that the majority of antibiotic research is still being conducted by small and medium-sized companies and in academia [5]. As a result, the outlook for human health appears grim, with recent estimates showing a likelihood of over 10 million deaths due to AMR by 2050 [6,7,8]. Although the number of novel antimicrobials has increased recently, most of these novel drugs do not harbor novel modes of action [5,9,10]. The persistent lack of innovation has led to fewer newly approved antibiotics in the pipeline than cancer drugs [11]. Certainly, incentives have to be developed to foster and enable development in this area [12,13]. Moreover, a recent report by the FDA has highlighted a number of key areas, including speeding up the development of new FDA-regulated products, reducing costs associated with assessing them, and improving health outcomes [14]. Thus, novel approaches will facilitate a faster transition from bench to bedside.

Once drugs enter the preclinical stage and, later, the clinical stage, pharmacokinetic (PK) and pharmacodynamic (PD) effects are investigated. However, this process is time consuming and expensive, in particular when entering clinical trials [5,12]. Therefore, it is of the utmost importance to understand PK/PD correlations and to estimate the probability of target attainment (PTA) in relevant compartments early in development. Traditionally, PK/PD considerations were based on the assumption that plasma concentrations are the best predictors of efficacy. This is certainly true and relevant in case of, e.g., bloodstream infections, but might have limitations for urinary tract infections, soft-tissue infections, or lung infections [15,16,17]. Recently, authorities have also attempted to demonstrate relevant concentrations in target tissues. Whereas the measurement of homogenized tissue concentrations neglects the compartmentalization of the tissue itself [18], approaches such as microdialysis or the determination of epithelial lining fluid (ELF) concentrations are more appropriate, but still have some restrictions, as the sampling of ELF in humans can be technically challenging [17,19,20,21,22,23,24,25]. The next step is to determine efficacy, i.e., the PD effect. Typically, this is performed using in vitro models, such as the minimal inhibitory concentration (MIC) [26], compartmental models [27], the determination of intracellularly active bacteria [28,29], time–kill curves in and without the presence of serum, and/or the hollow fiber infection model (HFIM) [30,31]; otherwise, it is performed using in vivo models, such as the neutropenic thigh, lung, sepsis, and urinary tract infection models [32,33,34,35,36,37,38]. These model systems serve to determine the so-called PK/PD index, a measure of efficacy depending on a certain PK parameter that is translatable from in vitro/preclinical species to humans. Most antibiotics fall into one of the three most clearly established categories: such as *f*C_max_/MIC, *f*AUC/MIC, or *f*T_>MIC_ [39]. Novel methods are needed to determine these PK/PD indices, in order to accelerate the process of demonstrating the target attainment of a drug. These novel methods can consist of deploying in silico methods to support or replace classical PK/PD determinations as performed with the currently available in vitro and in vivo systems [40,41,42,43,44,45].

The distinctiveness of the novel in silico methods lies in their diverse construction methods; this makes them a powerful tool capable of predicting target achievement. There are several options for constructing PK and PK/PD models [46,47]. Depending on the requirements and input data, rather simplistic compartmental models or more complex models, such as physiologically-based pharmacokinetic (PBPK) models, can be constructed [45,48,49,50,51,52]. The latter kind relies on the incorporation of physiological parameters and an organ-based substructure including sub-compartments. This enables the use of PBPK models inter alia for cross-species extrapolation and age-specific predictions of PK behavior [47,53]. Both types of model can then be linked to PD data, enabling to predict the probability of target attainment (PTA), i.e., a measure of how realistic a PD effect will be attained in vivo [54,55,56,57].

With this review, we aim to show how target attainment has been demonstrated by drugs that have been recently (less than five years) approved or drugs that are currently under late-stage clinical investigation. We selected drugs with different modes of action to highlight how PK/PD correlations and target attainment were determined in preclinical and clinical studies using different modelling techniques; we also investigated the lessons that can be learned for the development of novel anti-infectives. Our search criteria included the compound name, such as ‘tigecycline, omadacycline, ceftazidime, avibactam, gepotidacin, zoliflodacin and cefiderocol’, and the combined search terms ‘PK/PD’, ‘PK model’, ‘PK modeling’, ‘PK modelling’, and ‘PTA’.

## 2. Development of Ceftazidime/Avibactam: Using Modelling to Determine the PK/PD Target

Ceftazidime (CAZ) is a cephalosporin that is active against *P. aeruginosa*; together with avibactam (AVI), a non-ß-lactam ß-lactamase-inhibitor inhibiting enzymes belonging to Ambler classes A and C and selected class D beta-lactamases [58], it extends the spectrum against *Enterobacteriaceae* and several multi-drug resistant (MDR) *Pseudomonas* isolates [59,60,61]. In the last five years, novel beta-lactamase inhibitors, such as taniborbactam combined with a cephalosporin [62,63], as well as reports on the resistance of ceftazidime-avibactam (CAZ-AVI), have emerged [64,65]; however, we would like to highlight how the PK/PD targets were determined for AVI alone and how target attainment was achieved for the combination using PK/PD modelling. CAZ-AVI has been studied extensively in preclinical in vitro and in vivo models, as summarized elsewhere [66]. In the following section, we want to highlight some PK/PD modelling studies that contribute to a better understanding and prediction of PK/PD targets as well as PTA. A non-exhaustive list of those studies can be found in Table 1.

Determining a PK/PD target for a beta-lactamase inhibitor, such as AVI, can be quite challenging, as it does not harbor intrinsic bacterial activity. Thus, several studies deploy PK/PD modelling based on in vitro data and/or on in vivo data. A study by Sy et al. investigated the PK/PD correlations of CAZ-AVI by developing a mathematical model incorporating the bacterial killing and degradation of CAZ, in the case of absence of AVI and in the case of restoration of CAZ activity in presence of AVI [67]. This semi-mechanistic model was constructed for and reflected the PK/PD relationships of three different MDR *P. aeruginosa* isolates. Although only based on time–kill kinetic studies, i.e., studies under static conditions, it correctly predicted results from in vitro HFIM, i.e., studies under dynamic conditions, for several strains, similarly to previously published models [54,55,68,69]. Moreover, this model also correctly predicted log_10_ cfu reductions in neutropenic thigh and lung infection models conducted with more than 25 strains. This showed a successful validation of this model. Additionally, it demonstrated the power of properly validated PK/PD models: having a PK/PD model in place to predict PK/PD relationships for different strains in in vivo standard pharmacodynamics models helps to reduce animal experimentation and is completely in line with 3R. Impressively, the model produced by Sy and colleagues also predicted the log_10_ cfu change in patients for doses of 2 g CAZ and 0.5 g AVI as a 2 h infusion correctly [67]. Using this model, the same group was able to predict the PK/PD index for AVI as *f*T_>MIC_ [70] as it had previously been observed from experimental data [71,72]. Similarly to the predictions from Sy et al. [67,70], Kristofferson et al. constructed a semi-mechanistic model for *Enterobacteriaceae*, incorporating the scenarios and interplays of an actively growing susceptible bacterial population, a non-growing drug-insusceptible resting state, and a pre-existing mutant population [73]. In contrast to the models developed by Sy et al., this model incorporated a drug effect for AVI that had been observed in the initial stages of the time–kill curves. Thus, it described the effects of CAZ and AVI alone, as well as the ‘enhancer effect’ of AVI on CAZ. Again using simulated human dosing, the utility of the model was demonstrated. This suggests that, if properly validated, PK/PD models could also serve as an option for conducting clinical trials virtually in future, as observed in fields other than anti-infective research [74,75,76,77]; consequently, they may accelerate drug development.

**Table 1:** PK/PD models used for ceftazidime-avibactam.

Type of Model	Purpose	Reference
Compartmental PK/PD model	Prediction of PTA in vivo and in humans for *P. aeruginosa* and *Enterobacteriaceae*	[67,69,70,73]
Compartmental population PK/PD model	Estimation of PTA in adults for different indications	[78,79,80,81,82,83,84]

Nevertheless, there is another dimension to consider when using PK/PD models for reaching target attainment, especially in clinical practice. Whereas the aforementioned PK/PD models offer a perspective on which doses are needed to achieve a PD effect in a ‘standard’ human being, for clinical PK/PD models, covariates are explored, reflecting the characteristics of different populations, such as pediatric populations or populations in certain disease states, as frequently observed in infectious diseases. For this purpose, population PK/PD models were developed (PopPK/PD). In the PopPK/PD model constructed and validated by Li et al., the model aimed to assess certain factors, known as covariates, that influence the success of therapy, i.e., to calculate the PTA [80]. They constructed a two-compartment PopPK/PD model for adult populations in the indications of complicated intra-abdominal (cIAI) and complicated urinary tract infections (cUTI). They used nonlinear mixed-effects modelling (NONMEM) for healthy populations and patients from cIAI and cUTI phase III clinical trials and set the joint target for CAZ as 50% *f*T > 8 mg/L and for AVI as 50% *f*T > 1 mg/L, as described previously [72]. As both CAZ and AVI are mainly excreted via the kidneys, creatinine clearance was found to be one of the most predominant covariates, warranting dose adjustments in instances of creatinine clearance values lower than 50 mL/min. Thus, this study highlighted that, across indications (not only limited to cIAI and cUTI, but also including nosocomial pneumonia), the MIC breakpoint of 8 mg/L was sufficient to reach a PTA > 90% against *Enterobacteriaceae* and *P.aeruginosa*. Moreover, it gave guidance for dose adjustments in the case of renal insufficiency [80]. In addition to this study in adults, Franzese et al. addressed the question of PTA in pediatric patients older than three months [84]. They calculated the PTA based on the dosing regimens used in pediatric populations in previous clinical trials [85,86] and proved that the doses were sufficient to reach PTA in pediatric populations with normal renal function or mild renal impairment. Thus, the modelling results supported the current dosing scheme in clinical practice [84].

Another PopPK/PD model aimed to describe ELF concentrations, as ELF is the target compartment for pneumonia. Modelling ELF concentrations is useful, as the sampling of ELF concentrations is technically challenging. Therefore, Dimelow and colleagues constructed two- and three-compartment PK models to fit plasma concentration data and to be able to compare ELF levels to plasma PK/PD targets [79]. The study used ELF data from a previous phase I study [87] to build the models. It showed that the ELF:plasma ratios for CAZ and AVI were higher, especially at lower plasma concentrations. Thus, the study concluded that the ELF penetration was greater than that calculated previously using non-compartmental AUC methods. It demonstrated that, in instances of nonlinear drug penetration into the lung, as has been observed for CAZ and AVI, compartmental PopPK models can be useful and have broad applicability for drugs penetrating the lungs [79].

In conclusion, PK/PD modelling for CAZ-AVI did prove to be helpful in preclinical as well as clinical settings. To date, many of the studies involving CAZ-AVI have shown that, especially in the clinical context, modelling is of utmost importance and can be a tool for reducing the number of clinical trials needed to explore dosage regimens for specific populations and indications [78,88].

## 3. The Case of Omadacycline: PK/PD Modelling Data Still Scarce

Omadacycline (OMC) is an aminomethylcycline antibiotic, first approved in 2018 by the United States Food and Drug Administration (FDA). It belongs to the group of third-generation tetracyclines and, in contrast to tigecycline or eravacycline, it allows for intravenous as well as oral step-down therapy [89]. Like other tetracyclines, it binds to the 16S rRNA component of the 30S subunit and effectively blocks the aminoacyl-tRNA to the acceptor side of the ribosome in a reversible manner [90]. It has been demonstrated that, compared to other tetracyclines, OMC retains its activity in the instance of resistance mechanisms involving efflux pumps or ribosomal protection proteins [91,92]. However, recent studies suggest that cross-resistance might occur between OMC and, e.g., tigecycline [93].

In contrast to CAZ-AVI, there are scarce PK/PD modelling data for OMC to support current dosing regimens: only two PopPK models have been published so far [94,95]. This might be attributed to the fact that OMC was only approved recently (<five years). 

A study by Lakota et al. used a three-compartment model with first-order absorption and a transit compartment accounting for an absorption delay as a result of peroral administration and first-order elimination, as well as a three-compartment model with zero-order intravenous input; they gave the best fits upon validation with healthy subjects as well as with patients enrolled in phase 1b and phase 3 studies [94]. To enable the study of ELF levels, ELF concentrations were modelled as a sub-compartment of the first peripheral compartment. In that study, the model was constructed in such a way that OMC was distributed between the central (i.e., plasma) compartment and the first peripheral compartment. Moreover, ELF concentrations were estimated as a fraction of concentration in this first peripheral compartment. Using phase 3 patient data for external validation, population and individual plasma exposures were effectively captured. The timing of food consumption was considered an important factor, relative to dosing, for bioavailability. In the final model, this was parameterized as a function of the absolute time of food consumption relative to dosing by deploying a Hill-type function. Moreover, the assessment of patient-specific covariates showed the effect of sex was not of high importance, as C_max_ decreased only by 9%, C_min_ increased by 25%, and CL decreased by ~16% for females relative to males. In summary, the PopPK model described by Lakota et al. can serve as a good basis and starting point for subsequent PK/PD and PK/PD target attainment analysis [94].

It was surprising that only a few PK/PD models were available for OMC. A glance at the other third-generation tetracyclines shows that, for tigecycline, for example, only a few PK/PD modelling studies have been published to date [96,97,98]. As adjusted doses of tigecycline have been suggested for patients with severe hepatic functions [98], the only factor found in PK models for OMC affecting PK was the timing of food consumption. Future research might elucidate whether there are further covariates that should be considered for therapy with OMC, especially in critically ill patients. 

## 4. Gepotidacin and Zoliflodacin: Novel Bacterial Topoisomerase II Inhibitors under Clinical Development

Zoliflodacin (ZOL) was mainly developed to treat infections caused by *Neisseria gonorrhoeae* [99,100], although it is also active against *S. aureus* [99]. Gepotidacin (GEP) recently proved to be effective against *S. aureus*, *Streptococcus pneumoniae* and *Escherichia coli* [101,102,103], as well as a plethora of Gram-positive and Gram-negative anaerobes [104]. Additionally, both ZOL and GEP harbor novel chemical scaffolds as spiropyrimidinetriones and triazaacenaphthylenes, respectively. Due to their unique mechanism of action, ZOL and GEP have been assigned a new drug category, as “Novel Bacterial Topoisomerase Inhibitors” (NBTI). They are currently under phase 3 clinical investigation for uncomplicated gonorrhea (ZOL) and uncomplicated UTIs (GEP) [100,105].

Compared to fluoroquinolones that induce double-stranded DNA breaks, GEP induces breaks in single-stranded DNA [106], whereas ZOL binds to the GyrB subunit and inhibits DNA synthesis by the accumulation of double-stranded cleavages via the stabilization of the cleaved DNA [107]. Thus, both GEP and ZOL exhibit modes of action distinct from those of conventional fluoroquinolones, such as moxifloxacin, and thereby open up new areas for further investigation. Interestingly, the preclinical data indicate that GEP might not exert the same side-effects as conventional fluoroquinolones with respect to negative effects on skeletal development [108].

In contrast to OMC, several studies deployed PK/PD modelling. A list of those studies can be found in Table 2. However, in the following section, the techniques or specific outcomes of selected studies will be highlighted.

In a recent study that aimed to analyze the PK/PD relationships of ZOL in cases of suboptimal dosing and (partially) resistant strains of *N. gonorrhoeae*, a PopPK/PD model with three outputs, i.e., the concentration of ZOL, the total *N. gonorrhoeae* burden, and the burden of *N. gonorrhoeae* with lower susceptibility/resistance to ZOL, was developed [113]. Microbiological and in vitro PK/PD data for the *N. gonorrhoeae* strains upon treatment with different concentrations of ZOL were determined using an HFIM. Based on these in vitro data, the study aimed to predict the dosages to be used for therapy in humans that would enable eradication in a clinical setting. The results revealed that a single oral dose of 3 or 4 g eradicated a ZOL-susceptible *N. gonorrhoeae* strain and suppressed the amplification of selected mutants with increased ZOL MIC and *gyrB* resistance mutations. In contrast, 0.5, 1, and 2 g doses failed to eradicate it, resulting in selection for GyrB D429N-resistant populations. Moreover, the simulations revealed that ZOL double mutants (GyrB S467N plus the D429N substitution) might not be effectively treated, even with oral doses of 2–6 or 4–8 g q12h. Moreover, pre-existing ZOL-target GyrB S467N substitution was predisposed to develop resistance and the subsequent need for treatment with ZOL doses > 3 g. In conclusion, this study emphasized that rapid point-of-care testing might be necessary to detect possible *gyrB* mutations, as these have a high influence on the choice of ZOL dosing to assure successful therapy [113]. In a similar manner, Jacobsson and colleagues studied different WHO reference strains of *N. gonorrhoeae* in HFIM, using the same PopPK/PD model with three outputs [112]. Using this modelling, they were able to determine that the activity of ZOL was mainly concentration- rather than time-dependent. This finding was of particular importance for the design of subsequent clinical trials to enable the optimization of dosing compared to previous trials [114]. Moreover, they were able to demonstrate that the dosing frequency became less important for doses > 2 g of ZOL, as the kill rate *N. gonorrhoeae* approached a maximum and, consequently, supported the use of a single oral dose of 3 g of ZOL [112].

Likewise, several modelling studies have been conducted for GEP. Bulik and colleagues first conducted dose fractionation studies to determine the PK/PD index of GEP to inform clinical trials [39,115]. For GEP, *f*AUC/MIC best described the correlation of PK and PD. For the treatment of acute bacterial skin and skin structure infection (ABSSSI), a target *f*AUC/MIC ratio of 13.4 for *S. aureus* and of 14 for *S. pneumoniae* was identified; this was then implemented for the design of clinical trials [116]. Moreover, the study also assessed a post-antibiotic effect (PAE). The clinical utility of a PAE determined in vivo has been demonstrated previously [117]. Bulik and colleagues observed a PAE ranging from 3 to 12.5 h for *S. aureus* and from 5.25 to ~8.5 h for *S. pneumoniae* [115], which was comparable to fluoroquinolones [118]. As this effect was not dose dependent, the authors concluded that, in the case of GEP, the PAE was a predictable characteristic for informing decisions regarding clinical dosing intervals [115].

Hossain et al. developed a PBPK model to evaluate PK after the administration of different doses of GEP in populations without and with moderate or severe renal impairment [109]. Thereby, moderate renal impairment was defined with a GFR of 30 to 60 mL/min and severe renal impairment with a GFR of 15 to <30 mL/min. The rationale for choosing a PBPK model was that the researchers wanted to simulate GEP concentrations not only in plasma, but also in target compartments such as urine. Moreover, they also simulated concentrations in saliva, as the sampling of saliva might be more feasible when invasive blood sampling is not possible, e.g., in pediatrics. The PBPK model was verified with virtual healthy white and Japanese populations against available clinical PK data. They demonstrated that the C_max_ values increased while clearance decreased, with a higher severity degree of renal impairment. Saliva concentrations were linear to the observed plasma concentrations. However, the geometric mean AUC_0-t_ was elevated to a higher extent than plasma in all patient groups with renal impairment. Hemodialysis was not able to remove significant amounts of the drug from the system. Urine drug levels remained high, although a decrease was observed with decreasing renal function. The authors highlighted this fact as the urine concentrations were still in the target range for efficacy and, thus, important for the treatment of urinary tract infections. In summary, this study demonstrated the utility of PBPK modelling for GEP as it predicted not only plasma and urine concentrations, but also saliva concentrations with potential utility for sampling. 

In a similar manner, Nguyen et al. aimed to assess whether a PopPK or a PBPK model was best suited for predicting PK in pediatrics [110]. Both the PopPK and PBPK models were based on previously published models developed for adult populations. The PBPK model developed for adults was then used with similar parameters, but with pediatric physiology, including enzyme maturation. In contrast, the PopPK model was built using growth charts and tables for children that provided data for pediatrics aged from 2 to 20 years with the corresponding median body weights. Moreover, a second dataset from ModelRisk^®^ was used, accounting for the age and weight span for pediatrics from 0.01 to 12 years of age. Additionally, allometric exponents were used to account for size-related changes to clearance, as well as a maturation function accounting for size- and age-related changes to total clearance. Both models, the PopPK as well as the PBPK model, provided good predictions for GEP in pediatric populations. However, the authors concluded that the performance of the PopPK model in children aged three months and younger was suboptimal as a result of differences in the maturation characterization of the drug-metabolizing enzymes involved in clearance, which are unrelated to body weight in adults. Thus, this study showed the utility of PBPK modelling, as it might better account for special populations and also for disease populations because it has a mechanistic understanding of drug disposition.

In summary, modelling studies for ZOL and GEP were used to describe PK rather than to conduct PTA analysis. In contrast to, e.g. OMC, PBPK models were also used to better describe the distribution of drugs to different sub-compartments.

## 5. New Routes with Cefiderocol: From In Silico Studies to Market

In the face of rising carbapenem resistance [7], cefiderocol (CEF), a siderophore-conjugated cephalosporin, has recently emerged as a novel antibiotic with an unprecedented mode of action [119]. CEF was granted FDA approval in November 2019 and is used to treat cUTIs [120]. Unlike the structurally similar antibiotics ceftazidime and cefepime, CEF carries a chlorocatechol group on the end of the C-3 side chain, conferring siderophore activity [121,122,123]. With its siderophore properties [124,125], the drug enters the periplasm of bacteria, as it relies on active iron transport and maintains stability against ß-lactamases, thereby killing bacteria in a more efficient way [126,127].

Due to the fact that CEF was developed under the FDA streamline development program [128], which aims to minimize the number of clinical trials and instead relies primarily on preclinical evaluations of antimicrobial effectiveness, it is pertinent to focus on in silico strategies, such as PK/PD modelling. This section, therefore, outlines modelling studies conducted with regard to CEF to illustrate the innovative strategies being employed; we highlight their ability to help replenish the antibiotic pipeline in a more sustainable fashion (Table 3).

Kawaguchi and colleagues performed a series of studies on CEF utilizing compartmental modeling [129,130,131,132,134], with the most recent study utilizing an intrapulmonary PK model that adequately described the concentrations of the drug in ELF [132]. Data from 20 healthy subjects and 7 patients with pneumonia requiring mechanical ventilation were used to develop the model. They showed that the PTA values for both 75% *f*T_>MIC, ELF_ and 100% *f*T _>MIC, ELF_ were >90% against MICs ≤ 4 μg/mL in renally impaired patients and ≥87.0% in patients with normal renal function. Moreover, the delayed absorption and/or elimination of CEF in ELF was observed in patients with pneumonia but was not seen in healthy subjects, showing a difference in distribution towards ELF which might be attributed to the different physiological conditions in the lung, such as inflammation [25]. The authors concluded that the intrapulmonary PK modelling and PTA prediction were useful tools to support dosing recommendations in nosocomial pneumonia.

In a similar manner, Kawaguchi et al. aimed to conduct PTA analysis for different disease states, such as pneumonia, blood stream infection (BSI), sepsis, or cUTIs. Therefore, they built a PopPK/PD model to evaluate the impact of potential covariates on PK [131]. The PopPK model was constructed as a three-compartment model which accounted inter alia for the effects of creatinine clearance (CrCL), the effects of infection sites on total clearance (CL), the effect of body weight on the volume of distributions in the central and peripheral compartments, and albumin concentration. This more advanced PopPK model was built based on previous PopPK models for dose adjustment based on renal function [129] and on models evaluated with patient data from cUTI and acute uncomplicated pyelonephritis [130]. In this PopPK model, the estimated glomerular filtration rate (eGFR) adjusted by body surface area (BSA), absolute eGFR, and CrCL was used to calculate the plasma concentrations of CEF in PopPK [130]. Significant covariates in the final model were identified as the disease status (with or without infection) and body weight, although the effects of body weight were not considered to be clinically significant. The constructed model was able to effectively describe the plasma concentrations. Clear relationships between CL and all renal function markers were observed. Patients with infection had a 26% higher CL than those without infection. Notably, the authors found that CEF exposure in patients with infection was lower than in healthy subjects. However, in all patients with test regimens (2 g q8h as standard), the *f*T_>MIC_ values were higher than 75% (and, in most patients, 100%), suggesting sufficient exposure to CEF [130]. In the subsequent model, which included more disease states, such as BSI, pneumonia, and cUTI, they showed that CrCL was the dominant covariate [131]. The CL in patients with pneumonia, BSI, and cUTI was comparable to that of healthy subjects. Furthermore, they observed that C_max_ and AUC overlapped among infection sites and that they were similar in pneumonia patients with and without mechanical ventilation. With respect to PK/PD relationships, no clear correlation was found for any of the outcomes or the vital status, which might be attributed to the fact that the %*f*T_>MIC_ was 100% in most of the patients. Nevertheless, they calculated the 75% *f*T_>MIC,_ as well as the 100% *f*T_>MIC,_ for the simulated patients with different infection sites and renal functions. The PTA for 75% *f*T_>MIC_ was shown to be >95% against MICs < 4 μg/mL. This was independent of infection sites and renal function. In contrast, a difference was observed for PTA for 100% *f*T_>MIC_: here, the PTA was >90% against MICs < 4 μg/mL for all infection sites and renal function groups, except for normal renal function in BSI. In conclusion, this model demonstrated that, even with augmented renal clearance, PTA can be achieved with the recommended dosing regimens and adjusted regimens based on renal function in patients with pneumonia, BSI/sepsis, and cUTI caused by Gram-negative pathogens.

In summary, extensive modelling studies have been performed for CEF using different disease states and scenarios with respect to, e.g., the renal clearance. These studies demonstrate the power of PK/PD modelling as it helped to minimize clinical testing and to accelerate the development of CEF.

## 6. Discussion

We have discussed which specific models have been deployed for CAZ-AVI, OMC, GEP, ZOL, and CEF, and how they contribute to a better understanding of PK and PTA.

For CAZ-AVI several models, compartmental as well as PopPK models were only developed after approval; however, they still contribute to a better understanding of PK and PD and of dosing. It was especially surprising and encouraging how well predictions with respect to in vivo efficacy in animal models were made [69]. Moreover, in the case of CAZ-AVI, the PK/PD models helped to explain PK/PD indices for AVI and to estimate the dosages of AVI needed in combination with CAZ [73].

Surprisingly, published PK/PD modelling data for supporting dosing decisions and PTA are very scarce for OMC as well as tigecycline. Only PopPK models have been used; these require a fairly complex structure to capture all the characteristics of OMC after different routes of administration [94]. It is possible that, for OMC, this can be attributed to the fact that Lakota and colleagues only identified food consumption as an important covariate, but identified none of the conventional covariates, such as liver or renal function. Future studies for OMC might explain whether no other covariates influence PK.

In case of ZOL and GEP, compartmental, population and PBPK models were used. For the indications envisaged for ZOL and GEP, PBPK models are of particular interest as they enable researchers to model compound concentrations in target compartments and allow for a more complex structure. As shown here, the PBPK model enabled the prediction of saliva concentrations, which can be correlated with compound concentrations in other fluids; they therefore serve as a non-technically challenging sampling alternative [109]. The example of ZOL and GEP demonstrates that, dependent on the research question, either PopPK or PBPK models or both can be suitable [110].

Finally, several PopPK models of CEF demonstrated the power of validated PK/PD models. They served to predict PTA in different disease states and, adding slightly more complexity, for different degrees of renal function [129,130,131,132]. Notably, they did not deploy PBPK models, although this might have also enabled the prediction of compound concentrations in different target compartments, such as the lung or kidney. However, it seems that compartmental population models sufficed for prediction, meaning that there was no need for more complex models. The case of CEF demonstrates how modelling can help to determine dosages for different disease states and alterations in physiological functions in order to provide PTA and success of treatment.

## 7. Conclusions 

In conclusion, recently approved drugs as well as drugs in the later stages of development profit from PK/PD modelling, as it helps to elucidate PK, PK/PD, and which dose is needed for each disease to achieve a high PTA. Out of the drugs with the four different mechanisms of action presented in this review, CEF constitutes a prime example for accelerated development using PK/PD modelling, whereas the opposite seems to be true for OMC, at least with respect to the published modelling data. Finally, PK/PD modelling can help to reduce preclinical experimentation as well as clinical trials in the event that virtual clinical trials are conducted. These techniques are already frequently deployed in other disciplines, so that there is hope that they can also be used for the acceleration of antibiotic development to bring novel treatment options to patients and, ultimately, to help combat AMR.

## Figures and Tables

**Table 2:** PK and PK/PD models used for gepotidacin and zoliflodacin.

Type of Model	Purpose	Reference
PBPK	Prediction of GEP dosages in renally impaired patients	[109]
PBPK and PopPK	Prediction of GEP dose needed to treat pediatrics in case of plaque	[110]
PopPK	Prediction of dose and dose selection for GEP for a phase 3 study of the treatment of uncomplicated urogenital gonorrhea	[111]
Compartmental PK/PD model	Prediction of efficacious dose for ZOL that also suppresses resistance selection	[112,113]

**Table 3:** PK/PD models used for cefiderocol.

Type of Model	Purpose	Reference
Compartmental PopPK/PD model	Prediction for PTA in renally impaired patients and patients in different disease states	[129,130,131]
Compartmental PK	Prediction of exposure in ELF; prediction of total and unbound concentrations in critically ill patients	[132,133]

## Data Availability

No new data were created or analyzed in this study. Data sharing is not applicable to this article.

## References

[1] Laxminarayan R. (2022). The Overlooked Pandemic of Antimicrobial Resistance. Lancet.

[2] World Health Organization (2014). Antimicrobial Resistance: Global Report on Surveillance.

[3] Boucher H.W., Talbot G.H., Bradley J.S., Edwards J.E., Gilbert D., Rice L.B., Scheld M., Spellberg B., Bartlett J. (2009). Bad Bugs, No Drugs: No ESKAPE! An Update from the Infectious Diseases Society of America. Clin. Infect. Dis..

[4] Tacconelli E., Carrara E., Savoldi A., Harbarth S., Mendelson M., Monnet D.L., Pulcini C., Kahlmeter G., Kluytmans J., Carmeli Y. (2018). Discovery, Research, and Development of New Antibiotics: The WHO Priority List of Antibiotic-Resistant Bacteria and Tuberculosis. Lancet Infect. Dis..

[5] Theuretzbacher U., Outterson K., Engel A., Karlén A. (2020). The Global Preclinical Antibacterial Pipeline. Nat. Rev. Microbiol..

[6] de Kraker M.E.A., Stewardson A.J., Harbarth S. (2016). Will 10 Million People Die a Year Due to Antimicrobial Resistance by 2050?. PLoS Med..

[7] Murray C.J., Ikuta K.S., Sharara F., Swetschinski L., Robles Aguilar G., Gray A., Han C., Bisignano C., Rao P., Wool E. (2022). Global Burden of Bacterial Antimicrobial Resistance in 2019: A Systematic Analysis. Lancet.

[8] Ikuta K.S., Swetschinski L.R., Robles Aguilar G., Sharara F., Mestrovic T., Gray A.P., Davis Weaver N., Wool E.E., Han C., Gershberg Hayoon A. (2022). Global Mortality Associated with 33 Bacterial Pathogens in 2019: A Systematic Analysis for the Global Burden of Disease Study 2019. Lancet.

[9] Theuretzbacher U., Gottwalt S., Beyer P., Butler M., Czaplewski L., Lienhardt C., Moja L., Paul M., Paulin S., Rex J.H. (2019). Analysis of the Clinical Antibacterial and Antituberculosis Pipeline. Lancet Infect. Dis..

[10] Theuretzbacher U., Piddock L.J.V. (2019). Non-Traditional Antibacterial Therapeutic Options and Challenges. Cell Host Microbe.

[11] Walesch S., Birkelbach J., Jézéquel G., Haeckl F.P.J., Hegemann J.D., Hesterkamp T., Hirsch A.K.H., Hammann P., Müller R. (2023). Fighting Antibiotic Resistance—Strategies and (Pre)Clinical Developments to Find New Antibacterials. EMBO Rep..

[12] Miethke M., Pieroni M., Weber T., Brönstrup M., Hammann P., Halby L., Arimondo P.B., Glaser P., Aigle B., Bode H.B. (2021). Towards the Sustainable Discovery and Development of New Antibiotics. Nat. Rev. Chem..

[13] Piddock L.J.V., Paccaud J.-P., O’Brien S., Childs M., Malpani R., Balasegaram M. (2022). A Nonprofit Drug Development Model Is Part of the Antimicrobial Resistance (AMR) Solution. Clin. Infect. Dis..

[14] (2022). U.S. Food and Drug Administration Advancing Regulatory Cience at FDA: Focus Areas of Regulatory Science (FARS). https://www.fda.gov/media/161381/download.

[15] Müller M., dela Peña A., Derendorf H. (2004). Issues in Pharmacokinetics and Pharmacodynamics of Anti-Infective Agents: Distribution in Tissue. Antimicrob. Agents Chemother..

[16] Zhang D., Hop C.E.C.A., Patilea-Vrana G., Gampa G., Seneviratne H.K., Unadkat J.D., Kenny J.R., Nagapudi K., Di L., Zhou L. (2019). Drug Concentration Asymmetry in Tissues and Plasma for Small Molecule–Related Therapeutic Modalities. Drug Metab. Dispos..

[17] Jager N.G.L., van Hest R.M., Lipman J., Roberts J.A., Cotta M.O. (2019). Antibiotic Exposure at the Site of Infection: Principles and Assessment of Tissue Penetration. Expert Rev. Clin. Pharmacol..

[18] Mouton J.W., Theuretzbacher U., Craig W.A., Tulkens P.M., Derendorf H., Cars O. (2007). Tissue Concentrations: Do We Ever Learn?. J. Antimicrob. Chemother..

[19] Baldwin D.R., Wise R., Andrews J.M., Honeybourne D. (1991). Microlavage: A Technique for Determining the Volume of Epithelial Lining Fluid. Thorax.

[20] de la Peña A., Liu P., Derendorf H. (2000). Microdialysis in Peripheral Tissues. Adv. Drug Deliv. Rev..

[21] Joukhadar C., Derendorf H., Müller M. (2001). Microdialysis. Eur. J. Clin. Pharmacol..

[22] Joukhadar C., Müller M. (2005). Microdialysis. Clin. Pharmacokinet..

[23] Marchand S., Chauzy A., Dahyot-Fizelier C., Couet W. (2016). Microdialysis as a Way to Measure Antibiotics Concentration in Tissues. Pharmacol. Res..

[24] Heffernan A.J., Sime F.B., Sarovich D.S., Neely M., Guerra-Valero Y., Naicker S., Cottrell K., Harris P., Andrews K.T., Ellwood D. (2020). Pharmacodynamic Evaluation of Plasma and Epithelial Lining Fluid Exposures of Amikacin against Pseudomonas Aeruginosa in a Dynamic In Vitro Hollow-Fiber Infection Model. Antimicrob. Agents Chemother..

[25] Drwiega E.N., Rodvold K.A. (2022). Penetration of Antibacterial Agents into Pulmonary Epithelial Lining Fluid: An Update. Clin. Pharmacokinet..

[26] Landersdorfer C.B., Nation R.L. (2021). Limitations of Antibiotic MIC-Based PK-PD Metrics: Looking Back to Move Forward. Front. Pharmacol..

[27] VanScoy B.D., Lakota E.A., Conde H., Fikes S., Bhavnani S.M., Elefante P.B., Scangarella-Oman N.E., Ambrose P.G. (2021). Gepotidacin Pharmacokinetics-Pharmacodynamics against Escherichia Coli in the One-Compartment and Hollow-Fiber In Vitro Infection Model Systems. Antimicrob. Agents Chemother..

[28] Buyck J.M., Lemaire S., Seral C., Anantharajah A., Peyrusson F., Tulkens P.M., Van Bambeke F. (2016). In Vitro Models for the Study of the Intracellular Activity of Antibiotics. Methods Mol. Biol. Clifton NJ.

[29] Prochnow H., Fetz V., Hotop S.-K., García-Rivera M.A., Heumann A., Brönstrup M. (2019). Subcellular Quantification of Uptake in Gram-Negative Bacteria. Anal. Chem..

[30] McSharry J.J., Drusano G.L. (2011). Antiviral Pharmacodynamics in Hollow Fibre Bioreactors. Antivir. Chem. Chemother..

[31] Sadouki Z., McHugh T.D., Aarnoutse R., Ortiz Canseco J., Darlow C., Hope W., van Ingen J., Longshaw C., Manissero D., Mead A. (2021). Application of the Hollow Fibre Infection Model (HFIM) in Antimicrobial Development: A Systematic Review and Recommendations of Reporting. J. Antimicrob. Chemother..

[32] Hvidberg H., Struve C., Krogfelt K.A., Christensen N., Rasmussen S.N., Frimodt-Møller N. (2000). Development of a Long-Term Ascending Urinary Tract Infection Mouse Model for Antibiotic Treatment Studies. Antimicrob. Agents Chemother..

[33] Zhao M., Lepak A.J., Andes D.R. (2016). Animal Models in the Pharmacokinetic/Pharmacodynamic Evaluation of Antimicrobial Agents. Bioorg. Med. Chem..

[34] Andes D.R., Lepak A.J. (2017). In Vivo Infection Models in the Pre-Clinical Pharmacokinetic/Pharmacodynamic Evaluation of Antimicrobial Agents. Curr. Opin. Pharmacol..

[35] Drusano G.L. (2016). From Lead Optimization to NDA Approval for a New Antimicrobial: Use of Pre-Clinical Effect Models and Pharmacokinetic/Pharmacodynamic Mathematical Modeling. Bioorg. Med. Chem..

[36] Arrazuria R., Kerscher B., Huber K.E., Hoover J.L., Lundberg C.V., Hansen J.U., Sordello S., Renard S., Aranzana-Climent V., Hughes D. (2022). Expert Workshop Summary: Advancing toward a Standardized Murine Model to Evaluate Treatments for Antimicrobial Resistance Lung Infections. Front. Microbiol..

[37] Arrazuria R., Kerscher B., Huber K.E., Hoover J.L., Lundberg C.V., Hansen J.U., Sordello S., Renard S., Aranzana-Climent V., Hughes D. (2022). Variability of Murine Bacterial Pneumonia Models Used to Evaluate Antimicrobial Agents. Front. Microbiol..

[38] Rox K. (2022). Influence of Tramadol on Bacterial Burden in the Standard Neutropenic Thigh Infection Model. Sci. Rep..

[39] Ambrose P.G., Bhavnani S.M., Rubino C.M., Louie A., Gumbo T., Forrest A., Drusano G.L. (2007). Pharmacokinetics-Pharmacodynamics of Antimicrobial Therapy: It’s Not Just for Mice Anymore. Clin. Infect. Dis..

[40] Lodise T.P., Drusano G.L. (2014). Use of Pharmacokinetic/Pharmacodynamic Systems Analyses to Inform Dose Selection of Tedizolid Phosphate. Clin. Infect. Dis..

[41] Khan D.D., Friberg L.E., Nielsen E.I. (2016). A Pharmacokinetic–Pharmacodynamic (PKPD) Model Based on in Vitro Time–Kill Data Predicts the in Vivo PK/PD Index of Colistin. J. Antimicrob. Chemother..

[42] Sou T., Kukavica-Ibrulj I., Levesque R.C., Friberg L.E., Bergström C.A.S. (2020). Model-Informed Drug Development in Pulmonary Delivery: Preclinical Pharmacokinetic-Pharmacodynamic Modelling for Evaluation of Treatments against Chronic Pseudomonas Aeruginosa Lung Infections. Mol. Pharm..

[43] Sou T., Kukavica-Ibrulj I., Soukarieh F., Halliday N., Levesque R.C., Williams P., Stocks M., Cámara M., Friberg L.E., Bergström C.A.S. (2019). Model-Based Drug Development in Pulmonary Delivery: Pharmacokinetic Analysis of Novel Drug Candidates for Treatment of Pseudomonas Aeruginosa Lung Infection. J. Pharm. Sci..

[44] Sou T., Hansen J., Liepinsh E., Backlund M., Ercan O., Grinberga S., Cao S., Giachou P., Petersson A., Tomczak M. (2021). Model-Informed Drug Development for Antimicrobials: Translational PK and PK/PD Modeling to Predict an Efficacious Human Dose for Apramycin. Clin. Pharmacol. Ther..

[45] Rayner C.R., Smith P.F., Andes D., Andrews K., Derendorf H., Friberg L.E., Hanna D., Lepak A., Mills E., Polasek T.M. (2021). Model-Informed Drug Development for Anti-Infectives: State of the Art and Future. Clin. Pharmacol. Ther..

[46] Velkov T., Bergen P., Lora-Tamayo J., Landersdorfer C.B., Li J. (2013). PK/PD Models in Antibacterial Development. Curr. Opin. Microbiol..

[47] Rostami-Hodjegan A., Toon S. (2020). Physiologically Based Pharmacokinetics as a Component of Model-Informed Drug Development: Where We Were, Where We Are, and Where We Are Heading. J. Clin. Pharmacol..

[48] Bouzom F., Ball K., Perdaems N., Walther B. (2012). Physiologically Based Pharmacokinetic (PBPK) Modelling Tools: How to Fit with Our Needs?. Biopharm. Drug Dispos..

[49] Jones H.M., Mayawala K., Poulin P. (2013). Dose Selection Based on Physiologically Based Pharmacokinetic (PBPK) Approaches. AAPS J..

[50] Tsamandouras N., Rostami-Hodjegan A., Aarons L. (2015). Combining the ‘Bottom up’ and ‘Top down’ Approaches in Pharmacokinetic Modelling: Fitting PBPK Models to Observed Clinical Data. Br. J. Clin. Pharmacol..

[51] Ito M., Kusuhara H., Ose A., Kondo T., Tanabe K., Nakayama H., Horita S., Fujita T., Sugiyama Y. (2017). Pharmacokinetic Modeling and Monte Carlo Simulation to Predict Interindividual Variability in Human Exposure to Oseltamivir and Its Active Metabolite, Ro 64-0802. AAPS J..

[52] Lippert J., Burghaus R., Edginton A., Frechen S., Karlsson M., Kovar A., Lehr T., Milligan P., Nock V., Ramusovic S. (2019). Open Systems Pharmacology Community-An Open Access, Open Source, Open Science Approach to Modeling and Simulation in Pharmaceutical Sciences. CPT Pharmacomet. Syst. Pharmacol..

[53] Kuepfer L., Niederalt C., Wendl T., Schlender J., Willmann S., Lippert J., Block M., Eissing T., Teutonico D. (2016). Applied Concepts in PBPK Modeling: How to Build a PBPK/PD Model. CPT Pharmacomet. Syst. Pharmacol..

[54] Nielsen E.I., Viberg A., Löwdin E., Cars O., Karlsson M.O., Sandström M. (2007). Semimechanistic Pharmacokinetic/Pharmacodynamic Model for Assessment of Activity of Antibacterial Agents from Time-Kill Curve Experiments. Antimicrob. Agents Chemother..

[55] Nielsen E.I., Friberg L.E. (2013). Pharmacokinetic-Pharmacodynamic Modeling of Antibacterial Drugs. Pharmacol. Rev..

[56] Penney M., Agoram B. (2014). At the Bench: The Key Role of PK–PD Modelling in Enabling the Early Discovery of Biologic Therapies. Br. J. Clin. Pharmacol..

[57] Sadiq M.W., Nielsen E.I., Khachman D., Conil J.-M., Georges B., Houin G., Laffont C.M., Karlsson M.O., Friberg L.E. (2017). A Whole-Body Physiologically Based Pharmacokinetic (WB-PBPK) Model of Ciprofloxacin: A Step towards Predicting Bacterial Killing at Sites of Infection. J. Pharmacokinet. Pharmacodyn..

[58] Ehmann D.E., Jahić H., Ross P.L., Gu R.-F., Hu J., Durand-Réville T.F., Lahiri S., Thresher J., Livchak S., Gao N. (2013). Kinetics of Avibactam Inhibition against Class A, C, and D β-Lactamases. J. Biol. Chem..

[59] Sader H.S., Castanheira M., Mendes R.E., Flamm R.K., Farrell D.J., Jones R.N. (2015). Ceftazidime-Avibactam Activity against Multidrug-Resistant Pseudomonas Aeruginosa Isolated in U.S. Medical Centers in 2012 and 2013. Antimicrob. Agents Chemother..

[60] Nichols W.W., de Jonge B.L.M., Kazmierczak K.M., Karlowsky J.A., Sahm D.F. (2016). In Vitro Susceptibility of Global Surveillance Isolates of Pseudomonas Aeruginosa to Ceftazidime-Avibactam (INFORM 2012 to 2014). Antimicrob. Agents Chemother..

[61] de Jonge B.L.M., Karlowsky J.A., Kazmierczak K.M., Biedenbach D.J., Sahm D.F., Nichols W.W. (2016). In Vitro Susceptibility to Ceftazidime-Avibactam of Carbapenem-Nonsusceptible Enterobacteriaceae Isolates Collected during the INFORM Global Surveillance Study (2012 to 2014). Antimicrob. Agents Chemother..

[62] Yahav D., Giske C.G., Grāmatniece A., Abodakpi H., Tam V.H., Leibovici L. (2020). New β-Lactam–β-Lactamase Inhibitor Combinations. Clin. Microbiol. Rev..

[63] Satapoomin N., Dulyayangkul P., Avison M.B. (2022). Klebsiella Pneumoniae Mutants Resistant to Ceftazidime-Avibactam Plus Aztreonam, Imipenem-Relebactam, Meropenem-Vaborbactam, and Cefepime-Taniborbactam. Antimicrob. Agents Chemother..

[64] Bongiorno D., Bivona D.A., Cicino C., Trecarichi E.M., Russo A., Marascio N., Mezzatesta M.L., Musso N., Privitera G.F., Quirino A. (2023). Omic Insights into Various Ceftazidime-Avibactam-Resistant Klebsiella Pneumoniae Isolates from Two Southern Italian Regions. Front. Cell. Infect. Microbiol..

[65] Gaibani P., Gatti M., Rinaldi M., Crovara Pesce C., Lazzarotto T., Giannella M., Lombardo D., Amadesi S., Viale P., Pea F. (2021). Suboptimal Drug Exposure Leads to Selection of Different Subpopulations of Ceftazidime-Avibactam-Resistant Klebsiella Pneumoniae Carbapenemase-Producing Klebsiella Pneumoniae in a Critically Ill Patient. Int. J. Infect. Dis..

[66] Nichols W.W., Bradford P.A., Stone G.G. (2022). The Primary Pharmacology of Ceftazidime/Avibactam: In Vivo Translational Biology and Pharmacokinetics/Pharmacodynamics (PK/PD). J. Antimicrob. Chemother..

[67] Sy S.K.B., Zhuang L., Xia H., Beaudoin M.-E., Schuck V.J., Nichols W.W., Derendorf H. (2018). A Mathematical Model-Based Analysis of the Time–Kill Kinetics of Ceftazidime/Avibactam against Pseudomonas Aeruginosa. J. Antimicrob. Chemother..

[68] Bhagunde P., Chang K.-T., Hirsch E.B., Ledesma K.R., Nikolaou M., Tam V.H. (2012). Novel Modeling Framework To Guide Design of Optimal Dosing Strategies for β-Lactamase Inhibitors. Antimicrob. Agents Chemother..

[69] Sy S., Zhuang L., Xia H., Beaudoin M.-E., Schuck V., Derendorf H. (2017). Prediction of in Vivo and in Vitro Infection Model Results Using a Semimechanistic Model of Avibactam and Aztreonam Combination against Multidrug Resistant Organisms. CPT Pharmacomet. Syst. Pharmacol..

[70] Sy S.K.B., Zhuang L., Xia H., Schuck V.J., Nichols W.W., Derendorf H. (2019). A Model-Based Analysis of Pharmacokinetic–Pharmacodynamic (PK/PD) Indices of Avibactam against Pseudomonas Aeruginosa. Clin. Microbiol. Infect..

[71] Berkhout J., Melchers M.J., van Mil A.C., Seyedmousavi S., Lagarde C.M., Schuck V.J., Nichols W.W., Mouton J.W. (2015). Pharmacodynamics of Ceftazidime and Avibactam in Neutropenic Mice with Thigh or Lung Infection. Antimicrob. Agents Chemother..

[72] Nichols W.W., Newell P., Critchley I.A., Riccobene T., Das S. (2018). Avibactam Pharmacokinetic/Pharmacodynamic Targets. Antimicrob. Agents Chemother..

[73] Kristoffersson A.N., Bissantz C., Okujava R., Haldimann A., Walter I., Shi T., Zampaloni C., Nielsen E.I. (2020). A Novel Mechanism-Based Pharmacokinetic–Pharmacodynamic (PKPD) Model Describing Ceftazidime/Avibactam Efficacy against β-Lactamase-Producing Gram-Negative Bacteria. J. Antimicrob. Chemother..

[74] Toshimoto K., Tomaru A., Hosokawa M., Sugiyama Y. (2017). Virtual Clinical Studies to Examine the Probability Distribution of the AUC at Target Tissues Using Physiologically-Based Pharmacokinetic Modeling: Application to Analyses of the Effect of Genetic Polymorphism of Enzymes and Transporters on Irinotecan Induced Side Effects. Pharm. Res..

[75] Nakamura T., Toshimoto K., Lee W., Imamura C.K., Tanigawara Y., Sugiyama Y. (2018). Application of PBPK Modeling and Virtual Clinical Study Approaches to Predict the Outcomes of CYP2D6 Genotype-Guided Dosing of Tamoxifen. CPT Pharmacomet. Syst. Pharmacol..

[76] Sayama H., Marcantonio D., Nagashima T., Shimazaki M., Minematsu T., Apgar J.F., Burke J.M., Wille L., Nagasaka Y., Kirouac D.C. (2021). Virtual Clinical Trial Simulations for a Novel KRASG12C Inhibitor (ASP2453) in Non-Small Cell Lung Cancer. CPT Pharmacomet. Syst. Pharmacol..

[77] Yao B.-F., Wu Y.-E., Tang B.-H., Hao G.-X., Jacqz-Aigrain E., van den Anker J., Zhao W. (2022). Predictive Performance of Pharmacokinetic Model-Based Virtual Trials of Vancomycin in Neonates: Mathematics Matches Clinical Observation. Clin. Pharmacokinet..

[78] Bensman T.J., Wang J., Jayne J., Fukushima L., Rao A.P., D’Argenio D.Z., Beringer P.M. (2017). Pharmacokinetic-Pharmacodynamic Target Attainment Analyses To Determine Optimal Dosing of Ceftazidime-Avibactam for the Treatment of Acute Pulmonary Exacerbations in Patients with Cystic Fibrosis. Antimicrob. Agents Chemother..

[79] Dimelow R., Wright J.G., MacPherson M., Newell P., Das S. (2018). Population Pharmacokinetic Modelling of Ceftazidime and Avibactam in the Plasma and Epithelial Lining Fluid of Healthy Volunteers. Drugs RD.

[80] Li J., Lovern M., Green M.L., Chiu J., Zhou D., Comisar C., Xiong Y., Hing J., MacPherson M., Wright J.G. (2019). Ceftazidime-Avibactam Population Pharmacokinetic Modeling and Pharmacodynamic Target Attainment Across Adult Indications and Patient Subgroups. Clin. Transl. Sci..

[81] Kidd J.M., Stein G.E., Nicolau D.P., Kuti J.L. (2020). Monte Carlo Simulation Methodologies for β-Lactam/β-Lactamase Inhibitor Combinations: Effect on Probability of Target Attainment Assessments. J. Clin. Pharmacol..

[82] Li J., Lovern M., Riccobene T., Carrothers T.J., Newell P., Das S., Talley A.K., Tawadrous M. (2020). Considerations in the Selection of Renal Dosage Adjustments for Patients with Serious Infections and Lessons Learned from the Development of Ceftazidime-Avibactam. Antimicrob. Agents Chemother..

[83] Kang Y., Zhou Q., Cui J. (2021). Pharmacokinetic/Pharmacodynamic Modelling to Evaluate the Efficacy of Various Dosing Regimens of Ceftazidime/Avibactam in Patients with Pneumonia Caused by Klebsiella Pneumoniae Carbapenemase (KPC)-Producing, K. Pneumoniae: A Multicentre Study in Northern China. J. Glob. Antimicrob. Resist..

[84] Franzese R.C., McFadyen L., Watson K.J., Riccobene T., Carrothers T.J., Vourvahis M., Chan P.L.S., Raber S., Bradley J.S., Lovern M. (2022). Population Pharmacokinetic Modeling and Probability of Pharmacodynamic Target Attainment for Ceftazidime-Avibactam in Pediatric Patients Aged 3 Months and Older. Clin. Pharmacol. Ther..

[85] Bradley J.S., Broadhurst H., Cheng K., Mendez M., Newell P., Prchlik M., Stone G.G., Talley A.K., Tawadrous M., Wajsbrot D. (2019). Safety and Efficacy of Ceftazidime-Avibactam Plus Metronidazole in the Treatment of Children ≥3 Months to <18 Years With Complicated Intra-Abdominal Infection: Results From a Phase 2, Randomized, Controlled Trial. Pediatr. Infect. Dis. J..

[86] Bradley J.S., Roilides E., Broadhurst H., Cheng K., Huang L.-M., MasCasullo V., Newell P., Stone G.G., Tawadrous M., Wajsbrot D. (2019). Safety and Efficacy of Ceftazidime–Avibactam in the Treatment of Children ≥3 Months to <18 Years With Complicated Urinary Tract Infection: Results from a Phase 2 Randomized, Controlled Trial. Pediatr. Infect. Dis. J..

[87] Nicolau D.P., Siew L., Armstrong J., Li J., Edeki T., Learoyd M., Das S. (2015). Phase 1 Study Assessing the Steady-State Concentration of Ceftazidime and Avibactam in Plasma and Epithelial Lining Fluid Following Two Dosing Regimens. J. Antimicrob. Chemother..

[88] Das S., Li J., Riccobene T., Carrothers T.J., Newell P., Melnick D., Critchley I.A., Stone G.G., Nichols W.W. (2019). Dose Selection and Validation for Ceftazidime-Avibactam in Adults with Complicated Intra-Abdominal Infections, Complicated Urinary Tract Infections, and Nosocomial Pneumonia. Antimicrob. Agents Chemother..

[89] Zhanel G.G., Esquivel J., Zelenitsky S., Lawrence C.K., Adam H.J., Golden A., Hink R., Berry L., Schweizer F., Zhanel M.A. (2020). Omadacycline: A Novel Oral and Intravenous Aminomethylcycline Antibiotic Agent. Drugs.

[90] Chopra I. (1994). Tetracycline Analogs Whose Primary Target Is Not the Bacterial Ribosome. Antimicrob. Agents Chemother..

[91] Draper M.P., Weir S., Macone A., Donatelli J., Trieber C.A., Tanaka S.K., Levy S.B. (2014). Mechanism of Action of the Novel Aminomethylcycline Antibiotic Omadacycline. Antimicrob. Agents Chemother..

[92] Villano S., Steenbergen J., Loh E. (2016). Omadacycline: Development of a Novel Aminomethylcycline Antibiotic for Treating Drug-Resistant Bacterial Infections. Future Microbiol..

[93] Liu X., Zhang C., Zhao Y., Cheng H., Wang Y., Wang Z., Shang Y., Zheng J., Yu Z., Shi Y. (2022). Comparison of Antibacterial Activities and Resistance Mechanisms of Omadacycline and Tigecycline against Enterococcus Faecium. J. Antibiot..

[94] Lakota E.A., Van Wart S.A., Trang M., Tzanis E., Bhavnani S.M., Safir M.C., Friedrich L., Steenbergen J.N., Ambrose P.G., Rubino C.M. (2020). Population Pharmacokinetic Analyses for Omadacycline Using Phase 1 and 3 Data. Antimicrob. Agents Chemother..

[95] Yang H., Huang Z., Chen Y., Zhu Y., Cao G., Wang J., Guo Y., Yu J., Wu J., Liu L. (2022). Pharmacokinetics, Safety and Pharmacokinetics/Pharmacodynamics Analysis of Omadacycline in Chinese Healthy Subjects. Front. Pharmacol..

[96] Santimaleeworagun W., Hemapanpairoa J., Changpradub D., Thunyaharn S. (2020). Optimizing the Dosing Regimens of Tigecycline against Vancomycin-Resistant Enterococci in the Treatment of Intra-Abdominal and Skin and Soft Tissue Infections. Infect. Chemother..

[97] Zou D., Yao G., Shen C., Ji J., Ying C., Wang P., Liu Z., Wang J., Jin Y., Xiao Y. (2021). The Monte Carlo Simulation of Three Antimicrobials for Empiric Treatment of Adult Bloodstream Infections With Carbapenem-Resistant Enterobacterales in China. Front. Microbiol..

[98] Bastida C., Hernández-Tejero M., Cariqueo M., Aziz F., Fortuna V., Sanz M., Brunet M., Fernández J., Soy D. (2022). Tigecycline Population Pharmacokinetics in Critically Ill Patients with Decompensated Cirrhosis and Severe Infections. J. Antimicrob. Chemother..

[99] Bradford P.A., Miller A.A., O’Donnell J., Mueller J.P. (2020). Zoliflodacin: An Oral Spiropyrimidinetrione Antibiotic for the Treatment of Neisseria Gonorrheae, Including Multi-Drug-Resistant Isolates. ACS Infect. Dis..

[100] Unemo M., Ahlstrand J., Sánchez-Busó L., Day M., Aanensen D., Golparian D., Jacobsson S., Cole M.J., the European Collaborative Group (2021). High Susceptibility to Zoliflodacin and Conserved Target (GyrB) for Zoliflodacin among 1209 Consecutive Clinical Neisseria Gonorrhoeae Isolates from 25 European Countries, 2018. J. Antimicrob. Chemother..

[101] Biedenbach D.J., Bouchillon S.K., Hackel M., Miller L.A., Scangarella-Oman N.E., Jakielaszek C., Sahm D.F. (2016). In Vitro Activity of Gepotidacin, a Novel Triazaacenaphthylene Bacterial Topoisomerase Inhibitor, against a Broad Spectrum of Bacterial Pathogens. Antimicrob. Agents Chemother..

[102] Flamm R.K., Farrell D.J., Rhomberg P.R., Scangarella-Oman N.E., Sader H.S. (2017). Gepotidacin (GSK2140944) In Vitro Activity against Gram-Positive and Gram-Negative Bacteria. Antimicrob. Agents Chemother..

[103] Schuster S., Vavra M., Köser R., Rossen J.W.A., Kern W.V. (2021). New Topoisomerase Inhibitors: Evaluating the Potency of Gepotidacin and Zoliflodacin in Fluoroquinolone-Resistant Escherichia Coli upon TolC Inactivation and Differentiating Their Efflux Pump Substrate Nature. Antimicrob. Agents Chemother..

[104] Hackel M.A., Karlowsky J.A., Canino M.A., Sahm D.F., Scangarella-Oman N.E. (2022). In Vitro Activity of Gepotidacin against Gram-Negative and Gram-Positive Anaerobes. Antimicrob. Agents Chemother..

[105] Scangarella-Oman N.E., Hossain M., Hoover J.L., Perry C.R., Tiffany C., Barth A., Dumont E.F. (2022). Dose Selection for Phase III Clinical Evaluation of Gepotidacin (GSK2140944) in the Treatment of Uncomplicated Urinary Tract Infections. Antimicrob. Agents Chemother..

[106] Gibson E.G., Bax B., Chan P.F., Osheroff N. (2019). Mechanistic and Structural Basis for the Actions of the Antibacterial Gepotidacin against Staphylococcus Aureus Gyrase. ACS Infect. Dis..

[107] Basarab G.S., Kern G.H., McNulty J., Mueller J.P., Lawrence K., Vishwanathan K., Alm R.A., Barvian K., Doig P., Galullo V. (2015). Responding to the Challenge of Untreatable Gonorrhea: ETX0914, a First-in-Class Agent with a Distinct Mechanism-of-Action against Bacterial Type II Topoisomerases. Sci. Rep..

[108] Fishman C., Caverly Rae J.M., Posobiec L.M., Laffan S.B., Lerman S.A., Pearson N., Janmohamed S., Dumont E., Nunn-Floyd D., Stanislaus D.J. (2022). Novel Bacterial Topoisomerase Inhibitor Gepotidacin Demonstrates Absence of Fluoroquinolone-Like Arthropathy in Juvenile Rats. Antimicrob. Agents Chemother..

[109] Hossain M., Tiffany C., Raychaudhuri A., Nguyen D., Tai G., Alcorn H., Preston R.A., Marbury T., Dumont E. (2020). Pharmacokinetics of Gepotidacin in Renal Impairment. Clin. Pharmacol. Drug Dev..

[110] Nguyen D., Shaik J.S., Tai G., Tiffany C., Perry C., Dumont E., Gardiner D., Barth A., Singh R., Hossain M. (2022). Comparison between Physiologically Based Pharmacokinetic and Population Pharmacokinetic Modelling to Select Paediatric Doses of Gepotidacin in Plague. Br. J. Clin. Pharmacol..

[111] Scangarella-Oman N.E., Hossain M., Perry C.R., Tiffany C., Powell M., Swift B., Dumont E.F. (2023). Dose Selection for a Phase III Study Evaluating Gepotidacin (GSK2140944) in the Treatment of Uncomplicated Urogenital Gonorrhoea. Sex. Transm. Infect..

[112] Jacobsson S., Golparian D., Oxelbark J., Alirol E., Franceschi F., Gustafsson T.N., Brown D., Louie A., Drusano G., Unemo M. (2021). Pharmacodynamic Evaluation of Dosing, Bacterial Kill, and Resistance Suppression for Zoliflodacin Against Neisseria Gonorrhoeae in a Dynamic Hollow Fiber Infection Model. Front. Pharmacol..

[113] Jacobsson S., Golparian D., Oxelbark J., Franceschi F., Brown D., Louie A., Drusano G., Unemo M. (2022). Pharmacodynamic Evaluation of Zoliflodacin Treatment of Neisseria Gonorrhoeae Strains With Amino Acid Substitutions in the Zoliflodacin Target GyrB Using a Dynamic Hollow Fiber Infection Model. Front. Pharmacol..

[114] Taylor S.N., Marrazzo J., Batteiger B.E., Hook E.W., Seña A.C., Long J., Wierzbicki M.R., Kwak H., Johnson S.M., Lawrence K. (2018). Single-Dose Zoliflodacin (ETX0914) for Treatment of Urogenital Gonorrhea. N. Engl. J. Med..

[115] Bulik C.C., Okusanya Ó.O., Lakota E.A., Forrest A., Bhavnani S.M., Hoover J.L., Andes D.R., Ambrose P.G. (2017). Pharmacokinetic-Pharmacodynamic Evaluation of Gepotidacin against Gram-Positive Organisms Using Data from Murine Infection Models. Antimicrob. Agents Chemother..

[116] O’Riordan W., Tiffany C., Scangarella-Oman N., Perry C., Hossain M., Ashton T., Dumont E. (2017). Efficacy, Safety, and Tolerability of Gepotidacin (GSK2140944) in the Treatment of Patients with Suspected or Confirmed Gram-Positive Acute Bacterial Skin and Skin Structure Infections. Antimicrob. Agents Chemother..

[117] Craig W.A. (1993). Post-Antibiotic Effects in Experimental Infection Models: Relationship to in-Vitro Phenomena and to Treatment of Infections in Man. J. Antimicrob. Chemother..

[118] Craig W.A. (1998). Pharmacokinetic/Pharmacodynamic Parameters: Rationale for Antibacterial Dosing of Mice and Men. Clin. Infect. Dis..

[119] Kaye K.S., Naas T., Pogue J.M., Rossolini G.M. (2023). Cefiderocol, a Siderophore Cephalosporin, as a Treatment Option for Infections Caused by Carbapenem-Resistant Enterobacterales. Infect. Dis. Ther..

[120] Syed Y.Y. (2021). Cefiderocol: A Review in Serious Gram-Negative Bacterial Infections. Drugs.

[121] Sato T., Yamawaki K. (2019). Cefiderocol: Discovery, Chemistry, and In Vivo Profiles of a Novel Siderophore Cephalosporin. Clin. Infect. Dis..

[122] Mushtaq S., Sadouki Z., Vickers A., Livermore D.M., Woodford N. (2020). In Vitro Activity of Cefiderocol, a Siderophore Cephalosporin, against Multidrug-Resistant Gram-Negative Bacteria. Antimicrob. Agents Chemother..

[123] Simner P.J., Patel R. (2020). Cefiderocol Antimicrobial Susceptibility Testing Considerations: The Achilles’ Heel of the Trojan Horse?. J. Clin. Microbiol..

[124] Ji C., Juárez-Hernández R.E., Miller M.J. (2012). Exploiting Bacterial Iron Acquisition: Siderophore Conjugates. Future Med. Chem..

[125] Wilson B.R., Bogdan A.R., Miyazawa M., Hashimoto K., Tsuji Y. (2016). Siderophores in Iron Metabolism: From Mechanism to Therapy Potential. Trends Mol. Med..

[126] Ito A., Kohira N., Bouchillon S.K., West J., Rittenhouse S., Sader H.S., Rhomberg P.R., Jones R.N., Yoshizawa H., Nakamura R. (2016). In Vitro Antimicrobial Activity of S-649266, a Catechol-Substituted Siderophore Cephalosporin, When Tested against Non-Fermenting Gram-Negative Bacteria. J. Antimicrob. Chemother..

[127] Zhanel G.G., Golden A.R., Zelenitsky S., Wiebe K., Lawrence C.K., Adam H.J., Idowu T., Domalaon R., Schweizer F., Zhanel M.A. (2019). Cefiderocol: A Siderophore Cephalosporin with Activity Against Carbapenem-Resistant and Multidrug-Resistant Gram-Negative Bacilli. Drugs.

[128] Naseer S., Weinstein E.A., Rubin D.B., Suvarna K., Wei X., Higgins K., Goodwin A., Jang S.H., Iarikov D., Farley J. (2021). US Food and Drug Administration (FDA): Benefit-Risk Considerations for Cefiderocol (Fetroja^®^). Clin. Infect. Dis..

[129] Katsube T., Wajima T., Ishibashi T., Arjona Ferreira J.C., Echols R. (2016). Pharmacokinetic/Pharmacodynamic Modeling and Simulation of Cefiderocol, a Parenteral Siderophore Cephalosporin, for Dose Adjustment Based on Renal Function. Antimicrob. Agents Chemother..

[130] Kawaguchi N., Katsube T., Echols R., Wajima T. (2018). Population Pharmacokinetic Analysis of Cefiderocol, a Parenteral Siderophore Cephalosporin, in Healthy Subjects, Subjects with Various Degrees of Renal Function, and Patients with Complicated Urinary Tract Infection or Acute Uncomplicated Pyelonephritis. Antimicrob. Agents Chemother..

[131] Kawaguchi N., Katsube T., Echols R., Wajima T. (2021). Population Pharmacokinetic and Pharmacokinetic/Pharmacodynamic Analyses of Cefiderocol, a Parenteral Siderophore Cephalosporin, in Patients with Pneumonia, Bloodstream Infection/Sepsis, or Complicated Urinary Tract Infection. Antimicrob. Agents Chemother..

[132] Kawaguchi N., Katsube T., Echols R., Wajima T., Nicolau D.P. (2022). Intrapulmonary Pharmacokinetic Modeling and Simulation of Cefiderocol, a Parenteral Siderophore Cephalosporin, in Patients With Pneumonia and Healthy Subjects. J. Clin. Pharmacol..

[133] Zahr N., Urien S., Llopis B., Noé G., Tissot N., Bihan K., Junot H., Marin C., Mansour B., Luyt C.-E. (2022). Total and Unbound Pharmacokinetics of Cefiderocol in Critically Ill Patients. Pharmaceutics.

[134] Wenzler E., Butler D., Tan X., Katsube T., Wajima T. (2022). Pharmacokinetics, Pharmacodynamics, and Dose Optimization of Cefiderocol during Continuous Renal Replacement Therapy. Clin. Pharmacokinet..

